# 

**Published:** 1927

**Authors:** 


### The Medical Library of the University of Bristol,

WITH WHICH IS INCORPORATED

**The Library of the Bristol Medico-Chirurgical Society.**

---

*The following donations have been received since the publication of the list in May,* 1927.

September, 1927.

| | | |
|---|---|---|
| American Association of Genito-urinary Surgeons (1) .. .. .. .. .. | 1 | volume. |
| American Pediatric Society (2) .. .. | 1 | ,, |
| Carnegie Institution (3) .. .. .. | 1 | ,, |
| Dental Board of the United Kingdom (4) .. | 1 | ,, |
| Dr. L. M. Griffiths (bequest) (5) .. .. | 1 | ,, |
| Professor E. W. Hey Groves (6) .. .. | 2 | ,, |
| Johns Hopkins Press (7) .. .. .. | 1 | ,, |
| London School of Hygiene and Tropical Medicine (8) .. .. .. .. .. | 1 | ,, |
| Michell Clarke Memorial Fund (9) .. .. | 29 | ,, |
| Dr. G. Parker (10) .. .. .. .. | 1 | ,, |
| Rockefeller Foundation (11) .. .. .. | 1 | ,, |
| Dr. J. E. Shaw (12) .. .. .. .. | 2 | ,, |
| Dr. W. A. Smith (13) .. .. .. | 4 | ,, |
| Wiglesworth Bequest (14) .. .. .. | 8 | ,, |

Unbound periodicals have also been received from Professor E. W. Hey Groves.

### THE ONE HUNDRED AND TWENTY-FIRST LIST OF BOOKS.

The figures in round brackets refer to the figures after the names of the donors. The books to which no such figures are attached have either been bought from the Library Fund or received through the *Journal.*

**Baetjer, F. H., and Waters, C. A.** *Injuries and Diseases of the Bones and Joints* .. .. .. .. .. .. .. .. .. .. (9) 1921

**Bailey, P., and Cushing, H.** *Tumours of the Glioma Group* .. .. .. (9) 1926

**Ballenger, W. L.** .. *Diseases of the Nose, Throat and Ear* 5th Ed. (9) 1925

**Barjon, F.** .. .. .. *Radio-diagnosis of Pleuro-pulmonary Affections* (9) 1918

Bayly, H. W. .. .. *Venereal Disease* .. .. .. .. .. 3rd Ed. 1927
Belfrage, S. H. .. .. *What's Best to Eat?* .. .. .. .. .. .. .. .. 1926
Benedict, F. G., and Ritzman, E. G. *The Metabolism of the Fasting Steer* (3) 1927
Bertwistle, A. P., and Shenton, E. W. H. *Descriptive Atlas of Visceral Radiograms* .. .. .. .. .. .. .. .. .. .. (9) 1926
Bourne, A. W. .. .. *Synopsis of Midwifery and Gynæcology* 3rd Ed. 1925
Boyd, W. .. .. .. *Surgical Pathology* .. .. .. .. .. .. .. (9) 1925
Bradford, E. H., and Lovett, R. W. *Orthopedic Surgery* .. .. 5th Ed. (9) 1920
Brown, W. .. .. .. *Talks on Psychotherapy* .. .. .. .. .. .. (12) 1923
Buchanan, R. J. M. .. *Forensic Medicine and Toxicology (By J. E. W. MacFall)* .. .. .. .. .. .. .. .. .. (10) 1925
Burke, E. T. .. .. *Treatment of Venereal Disease* .. .. .. .. .. 1927
Buxton, P. A., and Hopkins, G. H. E. *Researches in Polynesia and Melanesia. Entomology* .. .. .. .. .. (8) 1927
Cabot, R. C... .. .. *Differential Diagnosis* .. Vol. II., 3rd Ed. (9) 1924
Camac, C. N. B... .. *Counsels and Ideals from the Writings of . . . W. Osler* .. .. .. .. .. .. .. .. .. (5) 1905
Cameron, S. J. .. .. *A Manual of Gynæcology* .. .. .. 3rd Ed. 1925
Cameron, S. J., & others *A Glasgow Manual of Obstetrics* .. .. .. .. .. 1924
Cawadias, A. P. .. .. *Diseases of the Intestines* .. .. .. .. .. (9) 1927
Cochrane, W. A. .. *Orthopedic Surgery* .. .. .. .. .. .. .. .. .. 1926
Cole, R. H. .. .. *Mental Diseases* .. .. .. .. .. 3rd Ed. (9) 1924
Coriat, I. H. .. .. *The Meaning of Dreams* .. .. .. .. .. .. (12) 1915
Craig, M., and Beaton, T. *Psychological Medicine* .. .. 4th Ed. (9) 1926
Dental Board of United Kingdom. *Dental Alloys* .. .. .. .. .. .. .. (4) —
Falconer, R. W. .. .. *The Bath Mineral Waters* .. .. .. .. .. .. 1861
Foote, J. A. .. .. *Diseases of the New-Born* .. .. .. .. .. (9) 1926
Geddes, G. .. .. .. *Puerperal Septicæmia* .. .. .. .. .. .. .. .. 1926
Graham, A. .. .. *Pathology and Treatment of Diabetes Mellitus* 2nd Ed. 1926
Guidott, T. .. .. .. *De Thermis Britannicis* .. .. .. .. .. .. .. .. 1691
Gulland, G. L., and Goodall, A. *The Blood* .. .. .. .. 3rd Ed. (9) 1925
Harris, W. .. .. .. *Neuritis and Neuralgia* .. .. .. .. .. .. .. (9) 1926
Hart, B. .. .. .. .. *Psychopathology* .. .. .. .. .. .. .. .. .. (9) 1927
Health of the Child of School Age. *By Various Authors* .. .. .. .. 1927
Hillary, W. .. .. *Lincomb Spaw Water* .. .. .. .. .. .. .. .. .. 1742
Howard, R., and Perry A. *The House-Surgeon's Vade-Mecum* 2nd Ed. 1926
Hurry, J. B. .. .. *Imhotep* .. .. .. .. .. .. .. .. .. .. .. .. 1926
Hutchison, R. .. .. *Lectures on Diseases of Children* .. 5th Ed. (9) 1925
Hutchison, R. .. .. *Elements of Medical Treatment* .. .. .. .. .. 1926
Irwin, W. K. .. .. *Urinary Surgery* .. .. .. .. .. .. 2nd Ed. 1927
Jelliffe, S. E., and White, W. A. *Diseases of the Nervous System* 4th Ed. (9) 1923
Jordan, A. C. .. .. *Chronic Intestinal Stasis* .. .. .. .. 2nd Ed. 1926
Kanavel, A. B. .. .. *Infections of the Hand* .. .. .. 5th Ed. (9) 1925
Keir, P. .. .. .. *Medicinal Waters of Bristol* .. .. .. .. .. (13) 1739
Lewis, W. B. .. .. *The Human Brain* .. .. .. .. .. .. .. .. (14) 1882
Lilienthal, H. .. .. *Thoracic Surgery* .. .. .. .. .. 2 Vols. (9) 1925
Lister Centenary Exhibition at the Wellcome Museum .. .. .. .. .. 1927
Lister and the Lister Ward at the Glasgow Royal Infirmary .. .. .. .. 1927

| | | | |
|---|---|---|---|
| **Lockyer, C.** | *Fibroids and Allied Tumours* | (9) | 1918 |
| **Low, R. C.** | *Anaphylaxis and Sensitization* | (9) | 1924 |
| **Lucas, C.** | *Bath and Bristol Waters* | (13) | [1764] |
| **Macalpine, J. B.** | *Cystoscopy* | | 1927 |
| **Mackenzie, Sir J.** | *Heart Disease and Pregnancy* | | 1921 |
| **MacNalty, A. S.** | *Epidemic Diseases of the Central Nervous System* | | 1927 |
| **Mickle, W. J.** | *Insanity* (*Goulstonian Lectures*) | (14) | 1888 |
| **Miles, A.** | *The Study of Medicine* | | 1925 |
| **Moore, R. F.** | *Medical Ophthalmology* | 2nd Ed. (9) | 1925 |
| **Mott, F. W.** | *Degeneration of the Neurone* (*Croonian Lecture*) | (14) | 1900 |
| **Mouchet, A., and Tavernier, L.** | *Pathologie des ménisques du genou* | (6) | 1927 |
| **Norris, E. W., and Landis, H. R. M.** | *Diseases of the Chest* | 3rd Ed. (9) | 1924 |
| **Oliver, W.** | *Bath Waters* | 4th Ed. | 1747 |
| **Pamphlets on Mineral Waters** | | | — |
| **Peirce, R.** | *Bath Memoirs* | | 1697 |
| **Randolph, G.** | *Enquiry into Medicinal Virtues of Bristol Water* | (13) | 1745 |
| **Raynaud, M.** | *Local Asphyxia.* (Trans. by. T. Barlow) | (14) | — |
| **Russell, E. H., and Russell, W. K.** | *Ultra-violet Radiation and Actinotherapy* | 2nd Ed. (9) | 1927 |
| **Sanderson, W., and Rayner, E. B. A.** | *The Law and Tradition of Medical Practice* | | 1926 |
| **Semon, R.** | *Mnemic Psychology* | (9) | 1923 |
| **Short, T.** | *History of the Mineral Waters of Derby, Lincs. and Yorks.* | | 1734 |
| **Sinclair, M.** | *The Thomas Splint* | | 1927 |
| **Spender, J. K.** | *The Bath Mineral Waters* | | 1877 |
| **Spry, J. H.** | *The Bath Waters* | | 1822 |
| **Stern, B. J.** | *Should We Be Vaccinated?* | | 1927 |
| **Stoddart, W. H. B.** | *Mind and its Disorders* | 5th Ed. (9) | 1926 |
| **Sutherland, A.** | *Bath and Bristol Waters* | 2nd Ed. (13) | 1764 |
| **Tuke, D. H.** | *History of the Insane in the British Isles* | (14) | 1882 |
| **Tweedy, E. H., Wrench, G. T., and Solomons, B.** | *Practical Obstetrics* | 5th Ed. | 1925 |
| **Underhill, J.** | *The Bristol Hot-Well Water* | | 1703 |
| **Walker, K. M.** | *The Enlarged Prostate* | (9) | 1926 |
| **Waring, H. J.** | *Manual of Operative Surgery* | 6th Ed. (6) | 1927 |
| **The Wellcome Historical Medical Museum** | | | 1927 |
| **Wilkinson, C. H.** | *The Bath Waters* | | 1811 |
| **Willow Bridge Waters, Staffordshire** | | | — |
| **Wyard, S.** | *Handbook of Diseases of the Stomach* | | 1927 |
| **Wynter, J.** | *Medicinal Waters of Bath and Bristol* | | 1728 |

### TRANSACTIONS, REPORTS, JOURNALS, ETC.

| | | |
|---|---|---|
| American Laryngological Association, Transactions | | 1926 |
| Asylums on the Continent. Report of Lancs. Asylums Board | (14) | 1900 |
| British Journal of Surgery. Vol. 15. No. 57 | | 1927 |
| Guy's Hospital Reports. Vol. 77. No. 2 | | 1927 |
| Johns Hopkins Hospital Reports. Vol. I. | (7) | 1896 |
| Journal of Mental Science. Vols. 61–63 | (14) | 1915–1917 |

| | | |
|---|---|---|
| Medical Annual, 1927 .. .. .. .. .. .. .. .. .. .. .. .. .. .. .. .. .. | | 1927 |
| Medical Society's Transactions. Vol. 49.. .. .. .. .. .. .. .. .. .. .. | | 1926 |
| Medicina Fennica, I and II. .. .. .. .. .. .. .. .. .. .. .. .. .. | | 1926–1927 |
| Methods and Problems of Medical Education. 7th Series .. .. .. .. | (11) | 1927 |
| Progressive Medicine. Vol. 2, 1927 .. .. .. .. .. .. .. .. .. .. .. | | 1927 |
| St. Bartholomew's Hospital Reports. Vol. 60 .. .. .. .. .. .. .. .. | | 1927 |
| Surgical Clinics of North America. Vols. I. and II. (12 vols.) .. | (9) | 1921–1922 |
| Transactions of American Association of Genito-Urinary Surgeons. Vols. 11, 17, 19 .. .. .. .. .. .. .. .. .. .. .. .. .. | (1) | 1916, 1924, 1926 |
| Transactions of American Pediatric Society. Vols. 37, 38 .. | (2) | 1926–1927 |
| Transactions of International Medical Congress. 4 Vols. .. .. .. | (14) | 1881 |
| Virchows Archiv. Vols. 217–225, 227 .. .. .. .. .. .. .. .. | (9) | 1914–1920 |

### Local Medical Notes.

EXAMINATION RESULTS.

**University of Bristol.**—Students of the University have recently passed the following examinations :—

M.B., Ch.B.—*First Examination. Pass :* G. T. Bevir, W. R. Cambridge, C. J. N. Davis, H. F. M. Finzel, F. E. Fletcher, J. R. Gibbs, H. James, C. N. Royal, Frances Norma Salisbury.

M.B., CH.B. (*Part I.*).—*Second Examination. Pass :* D. P. Dewe, R. H. Moore. *In Organic Chemistry* (*Completing Examination*) *:* J. L. S. Coulter, W. A. F. Taylor. *In Elementary Anatomy* (*Completing Examination*) *:* L. C. Holland, Elizabeth S. G. Owen. *In Elementary Anatomy only :* C. J. N. Davis, C. N. Royal.

F.R.C.S.—*Primary Examination. Pass :* H. E. Harris, M.R.C.S., L.R.C.P., G. F. Langley, S. C. Wake.

M.R.C.S., L.R.C.P.—J. T. Chesterman, H. L. Fuller, J. C. Gent.

L.D.S., R.C.S.—A. F. D. Shapland.

L.S.A.—*Surgery* (*Completing Course for the Diploma*) *:* S. K. Rigg. *Materia Medica and Pharmacy :* T. A. Barnabas. *Physiology :* J. S. Adamson, F. W. A. Fosbery.

